# Using the transient trajectories of an optically levitated nanoparticle to characterize a stochastic Duffing oscillator

**DOI:** 10.1038/s41598-020-70908-z

**Published:** 2020-09-02

**Authors:** Jana Flajšmanová, Martin Šiler, Petr Jedlička, František Hrubý, Oto Brzobohatý, Radim Filip, Pavel Zemánek

**Affiliations:** 1 Institute of Scientific Instruments of the Czech Academy of Sciences, Královopolská 147, 612 64 Brno, Czech Republic; 2 Department of Optics, Palacký University, 17. listopadu 1192/12, 771 46 Olomouc, Czech Republic

**Keywords:** Optics and photonics, Optical manipulation and tweezers, Quantum mechanics

## Abstract

We propose a novel methodology to estimate parameters characterizing a weakly nonlinear Duffing oscillator represented by an optically levitating nanoparticle. The method is based on averaging recorded trajectories with defined initial positions in the phase space of nanoparticle position and momentum and allows us to study the transient dynamics of the nonlinear system. This technique provides us with the parameters of a levitated nanoparticle such as eigenfrequency, damping, coefficient of nonlinearity and effective temperature directly from the recorded transient particle motion without any need for external driving or modification of an experimental system. Comparison of this innovative approach with a commonly used method based on fitting the power spectrum density profile shows that the proposed complementary method is applicable even at lower pressures where the nonlinearity starts to play a significant role and thus the power spectrum density method predicts steady state parameters. The technique is applicable also at low temperatures and extendable to recent quantum experiments. The proposed method is applied on experimental data and its validity for one-dimensional and three-dimensional motion of a levitated nanoparticle is verified by extensive numerical simulations.

## Introduction

Linear harmonic oscillators are useful idealisations explaining a broad class of phenomena. However, real oscillators are always nonlinear. Typically, they exhibit a soft Duffing nonlinearity turning oscillations to anharmonic one. Despite long-term experimental investigation, new diverse effects have been recently observed in the underdamped Duffing oscillators based on nano-electromechanical systems^1–3^, micro-electromechanical systems^4–8^, nonlinear electric oscillators^9^, particles trapped in nonlinear potentials^10^, solid-state systems^11^, mechanical oscillators with a chemical bond^12^ and also proposed for upcoming quantum mechanical oscillators with superconducting qubits^13^. They stimulate further investigations of both equilibrium states and transient dynamics of anharmonic oscillators.

At long time scales, when dynamics tends to equilibrate, and for short transient times, the anharmonicity can have different impacts. A precise description of transient effects in nonlinear oscillators is therefore essential for our understanding of nonequilibrium physics and applications ranging from nanosensing and thermodynamically engines up to social dynamics^14–23^. The faithful characterization of a nonlinear oscillator requires to estimate its parameters beyond standard methodology based either on the equilibrium state (ES) or on the power spectral density (PSD) of particle positions or velocities^24–29^. Both these methods presume that values estimated from steady states are valid also during the transient dynamics. Such assumption can, however, lead to significant systematic errors in the values of estimated parameters, e.g. in the case of a nonlinear oscillator with large amplitudes, as we demonstrate in this paper.

Currently, optomechanics with a levitating particle oscillating in a nonlinear potential formed by an electromagnetic field in an optical or radiofrequency spectral region is a viable platform to test and understand many new nonlinear phenomena and, ultimately, bring them to very low temperatures where quantum mechanics affects the oscillations^28, 30–37^. This trapping technique provides us with new possibilities for mechanical sensing and manipulation in the field of nanotechnology and chemistry. It is therefore desirable that the methodology characterizing parameters of a nonlinear oscillator is applicable also in quantum mechanics. Moreover, the methodology should apply to transient effects, in contrast to the widespread approach based on PSD.

The levitating nanoparticle system is specific because the nanoparticle position is typically the only directly observable quantity. The velocity can be in principle determined using an independent Doppler velocimetry^38^ or by homodyne detection^39^ but this technique requires a high speed detection and data acquisition system. An object velocity or momentum is most frequently estimated by a numerical differentiation of measured positions of a levitating particle. Therefore, a method which uses information mainly from the particle position is generally more broadly applicable.

In this paper, we present a suitable approach estimating all desired parameters of a weakly nonlinear Duffing oscillator assuming that the particle radius is known. Our estimation method is based on a post-processing of acquired time records of particle positions and averaging trajectories in the statistical ensemble. We compare our results with the commonly used PSD method and, unlike Refs.^18, 40^, we obtain the Duffing coefficient of nonlinearity without any external driving force which could affect the system parameters and would be undesirable for experimental studies of quantum effects. Moreover, the determined damping coefficient follows well the theoretical prediction down into low pressures. Our method is capable of the determination of parameters on the time scale that is faster than the heating rate which is crucial for experimental testing of transient stochastic phenomena with levitating particles.

## Experimental set-up and data processing

**Figure 1:**
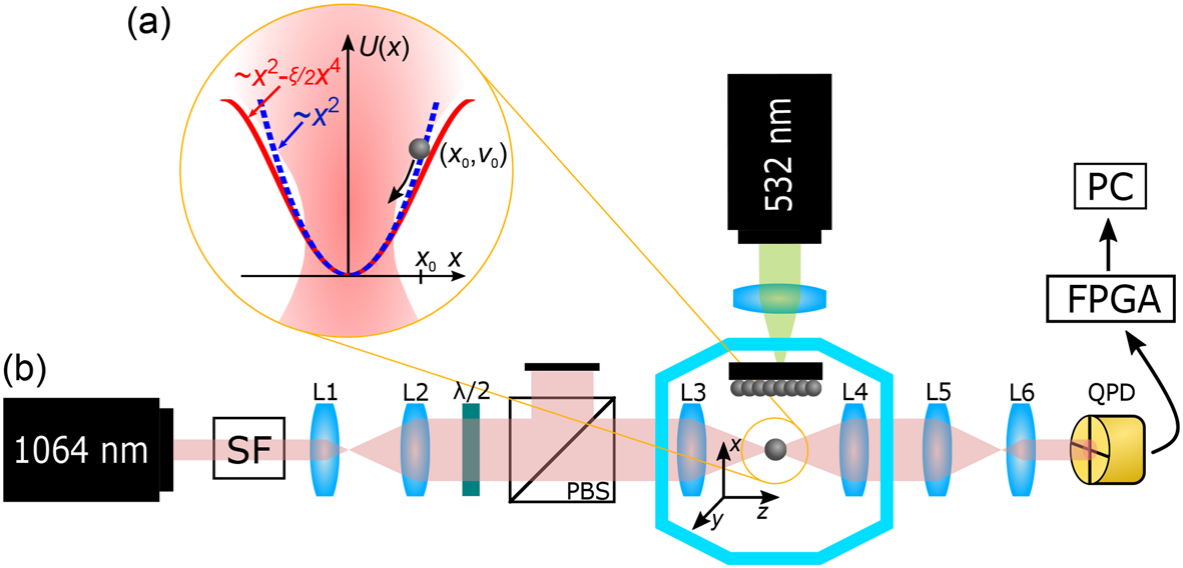
(**a**) A scheme of a nanoparticle (NP) levitating in one-dimensional optical potential along the lateral axis $x$. Dashed blue and solid red curves denote the parabolic shape of the ideal harmonic oscillator and nonlinear Duffing potential, respectively. Initial conditions of the particle motion are denoted as $\left({x}_{0},{v}_{x0}\right)$. (**b**) Experimental set-up. A low-noise $y$-polarized laser beam (wavelength 1064 nm, Mephisto 2000NE) is $3\times$ expanded using lenses L1 and L2 (Thorlabs AC127-025-C, AC254-075-C). The trapping power is controlled by a rotating half-wave plate $\lambda /$2 (Thorlabs WPH10M-1064) placed in front of a polarizing beamsplitter PBS (Thorlabs PBS253). The beam is focused by an aspheric lens L3 (Lightpath 355330, NA = 0.77) placed inside a vacuum chamber and maximal beam power 100 mW can be obtained here. Silica NPs (170 nm in diameter, Bangs Laboratories, Inc.) are launched from a silicon substrate towards the beam focus by a focused laser pulse (wavelength 532 nm, energy 4 mJ in a single pulse, Continuum Minilite MLII)^60^ under a chamber pressure of 7 mBar. The unscattered and scattered light by the NP trapped near the beam focus is collected by an aspheric lens L4 (Thorlabs C240-TME) in forward direction, demagnified by a telescope L5 and L6 (Thorlabs AC254-250-C, AC254-030-C), and detected by a quadrant photodetector QPD (Hamamatsu G6849). The QPD signal is processed by a home-made electronics and the NP positions in all three axes are acquired by National Instruments FPGA card (NI FPGA NI 5783 adapter module, FlexRIO FPGA) with the sampling frequency 1.78 MHz.

A nanoparticle (NP) is trapped in a focused laser beam in all three orthogonal directions. A scheme of an optical trap in a transversal direction is shown in Fig. 1a. Since the non-conservative scattering force is proportional to a sphere radius $a$ as ${a}^{6}$^41, 42^, it can be neglected with respect to the conservative gradient forces acting upon an NP. Thus the spatial profile of the trapping potential $U\left(x,y,z\right)$ follows an inverted shape of the optical intensity $I\left(x,y,z\right)$ as $U\left(x,y,z\right)\simeq -I\left(x,y,z\right)$. Since the real lateral or longitudinal intensity profile is close to the Gaussian or Lorentzian shape^43^, respectively, the potential energy of the NP displaced further from the equilibrium position is lower in the real optical trap comparing to the ideal parabolic potential (compare solid red and dashed blue curves in Fig. 1a). Such slight deviations from an ideal shape are frequently neglected for an NP with cooled translational motion^28^ because it moves in the dominantly parabolic bottom of the potential well, and the NP trajectory is analyzed following the theory for an ideal harmonic oscillator^29^. However, if NP’s motion is cooled but coherently excited, the nonlinearities in the potential arise and drag the NP motion out of ideal harmonic oscillations. It is the same nonlinearity affecting the NP at higher temperatures. Assuming just the first lower-even-order terms in Taylor series expansion of the real profile of the potential near its minimum^18, 44^, see also Supplementary Information, it gives the Duffing type nonlinearity. The nonlinearity in the system can be characterized by the stiffness of the Duffing spring that depends on the position $x$ of the object as $\kappa_{{\text{D}}} = \kappa \left( {1 - \xi x^{2} } \right)$, where $\kappa$ is the stiffness of the ideal harmonic oscillator and $\xi$ represents the coefficient of Duffing nonlinearity. In the real optical traps the coefficient $\xi$ is positive and thus the stiffness of the optical spring gets weaker with an increasing deviation from the equilibrium position $x = 0$. Such an oscillator is called a softening Duffing oscillator^14, 18, 45, 46^. Technical details of the whole experimental set-up are described in Fig. 1b.

**Figure 2:**
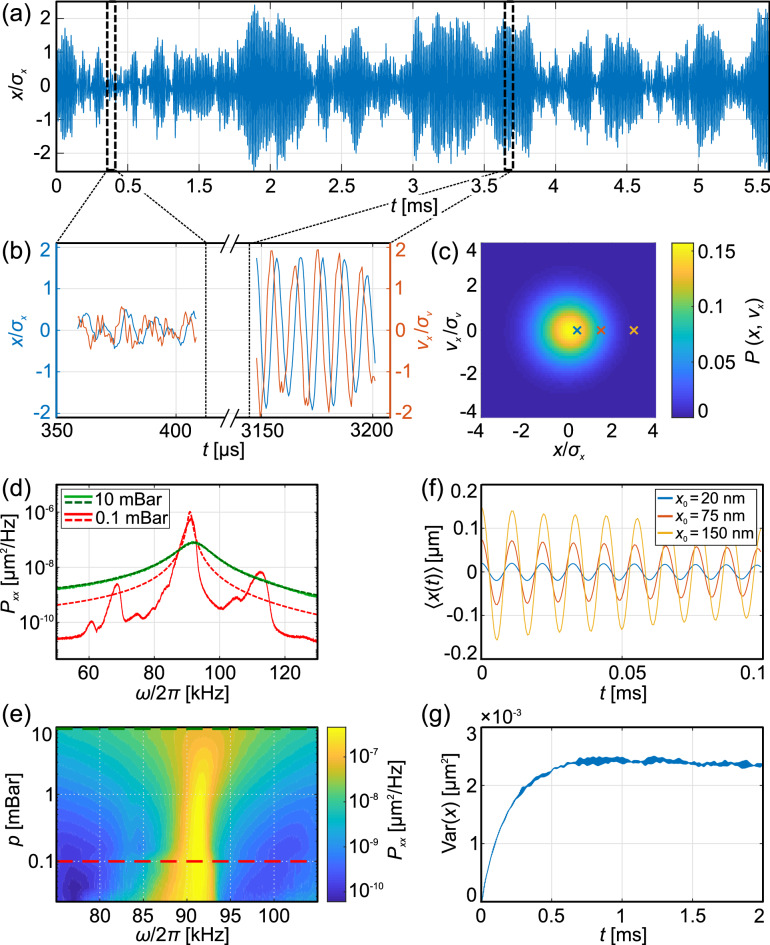
(**a**) Particle trajectory in the $x$ axis normalized to its standard deviation $\sigma_{x}$ for the ambient pressure 1 mBar. (**b**) Magnified regions of the trajectory (blue) from (**a**) for small and large oscillations together with the corresponding velocity (orange) obtained by the central difference normalized to its standard deviation $\sigma_{v}$. (**c**) Probability density function of the NP in the phase space at the ambient pressure 1 mBar for a 30 s long data set. Cross marks define starting points of trajectories shown in (**f**). (**d**) Power spectral density (PSD) of *x* positions of a trapped NP (full curves) with fitted functions given by Eq. (1) (dashed curves) for two different pressures. Obtained frequency and damping are ${\Omega }_{0} /\left( {2\pi } \right) = \left( {92403.0 \pm 0.4} \right)$ Hz, ${\Gamma } = \left( {59367 \pm 9} \right)$ s^−1^ and ${\Omega }_{0} /\left( {2\pi } \right) = \left( {91054 \pm 3} \right)$ Hz, ${\Gamma } = \left( {14810 \pm 70} \right)$ s^−1^ for pressures $p = 10$ and $p = 0.1$ mBar, respectively. (**e**) Two-dimensional map showing measured PSD evolution for different pressures. The green and red dashed lines correspond to the pressure 10 mBar and 0.1 mBar shown in (**d**), respectively. (**f**) Mean trajectories obtained by averaging the trajectory sections starting at the same point in the phase space $\left( {x_{0} ,0} \right)$ for different initial amplitudes $x_{0}$ at pressure 1 mBar. (**g**) An averaged variance ${\text{Var}}\left( x \right)$ for trajectories started at the same point in the phase space $\left( {0, 0} \right)$ at pressure 1 mBar.

An example of a 1D trajectory of an optically levitated NP in a potential well formed by a single strongly focused Gaussian laser beam is plotted in Fig. 2a. It shows that the NP oscillates with an amplitude that varies in time. Figure 2b shows two magnified regions of Fig. 2a (blue) and corresponding NP instantaneous velocity (orange) determined as a central difference $v_{x} \left( t \right) = \left[ {x\left( {t + {\Delta }t} \right) - x\left( {t - {\Delta }t} \right)} \right]/2{\Delta }t$, where ${\Delta }t$ is the time step in data acquisition given by the sampling frequency ${\Delta }t = 1/f_{{{\text{sample}}}} = 1/1.78$ μs which is sufficiently high comparing to the examined processes (~ 90 kHz). In the first example, the NP “wiggles” with a low amplitude ($\left| x \right| < {\upsigma }_{x}$, where $\sigma_{x}$ is the standard deviation of the particle position) close to the bottom of the potential (center of the optical trap) while the second part of the trajectory shows the sustained oscillations with a higher amplitude ($\left| x \right| > \sigma_{x}$). At the same time scale, small-amplitude motion is strongly affected by noise whereas the large-amplitude oscillations are rather modified by the nonlinear potential softening. Furthermore, Fig. 2c shows a two dimensional histogram of the Gaussian shape obtained from a long-time data set (30 s) showing probability density function of an NP being in a given volume of a phase space—i.e. having given position and velocity in the $x$ axis.

A standard steady-state analysis of NP motion in a harmonic potential is based on the power spectral density (PSD) of NP positions described by the following function^10, 24, 26, 28, 47, 48^

1 $$P_{xx} \left( \omega \right) = \frac{{2k_{{\text{B}}} T_{{{\text{SP}}}} }}{m}\frac{\Gamma }{{\left( {\omega^{2} - \Omega_{0}^{2} } \right)^{2} + \Gamma^{2} \omega^{2} }} + P_{{\text{s}}} ,$$

where $k_{{\text{B}}}$, $T_{{{\text{SP}}}}$, $m$, $\Omega_{0} = 2\pi f_{0}$, $\omega$, $\Gamma$, and $P_{{\text{s}}}$ denote the Boltzmann constant, effective spectral temperature of the thermal bath driving an NP with a white noise distribution, particle mass, NP eigenfrequency (in rad/s), frequency, medium damping, and technical measurement noise^29^, respectively. PSDs calculated from the long data set for two different ambient pressures are shown in Fig. 2d. For ambient pressure of 10 mBar and higher we obtained almost perfect fit of Eq. (1) to experimental data because the peak broadening is caused predominantly by the damping $\Gamma$ and the resonant peak is not visibly influenced by the nonlinear behaviour or crosstalks with other axes. In contrast in the lower pressures noticeable differences appear: (1) the measured peak is wider and lower than the theoretically predicted one, (2) there is a clear asymmetry of this peak and it exhibits a visible negative skewness. Such a deviation of the experimental PSD profile from the ideal one can be caused by the softening of the experimental potential profile from the assumed ideal harmonic trap^18, 29, 44, 49^. If we consider the lowest order of nonlinearity characteristic for a Duffing oscillator (see Fig. 1a), the oscillation frequency depends on the amplitude of oscillations^18, 44, 45^ as experimental data in Fig. 2f demonstrate. This behaviour leads to a continuum of new frequency components that contribute to the PSD at frequencies below the PSD resonant frequency (main peak) and cause asymmetry of the PSD peak observed in Fig. 2d for the lower pressure^49^. The secondary peaks observed in Fig. 2d are caused by crosstalks between the measured $x$ axis and other coordinate axes $y$ and $z$ and their higher harmonics. Figure 2e reveals experimental evolution of the main peak for more values of the ambient pressure with two highlighted levels of pressure discussed above.

Obviously, fitting the steady-state PSD of a real nonlinear oscillator with Eq. (1), which is valid only for the ideal harmonic oscillator, has to lead to an imperfect determination of parameters of the real oscillator^29^, as we show later in Fig. 5. Moreover, this PSD approach is not suitable for current investigation and use of transient out-of-equilibrium coherent effects faster than any heating of the motion^40^. After the cooling of levitating systems to the ground state^37^, such estimation of the Duffing oscillator from fast transient effects are crucial for upcoming studies of quantum effects^50–54^.

Therefore, in this work, we propose a novel method for determination of parameters of an optically levitated NP. Unlike the methods based on equipartition theorems in equilibrium steady-state or PSD methods that are independent of initial conditions, we post-process measured stochastic trajectories for selected suitable initial conditions and follow an NP trajectory during a short transient dynamics to determine required parameters. However, we study a stochastic system where a random noise influences the individual trajectories of the NP probing a nonlinear potential. Thus each transient process starting at the same point in the phase space $\left( {x_{0} ,v_{x0} } \right)$ leads to a different trajectory. Therefore, we opted for an analysis of the moments (e.g. mean and variance) of such ensemble of NP trajectories that start in the same volume of the phase space. Statistical moment dynamics can be further used up to the ground state of mechanical motion.

The consequent post-processing algorithm for one axis (shown for the $x$ axis) proceeds in the following steps:

1. Record of QPD signals in voltage is transformed to the NP positions $x\left( t \right)$ by the calibration factor $C_{{{\text{V}} \to {\text{m}}}}$ described in Methods.
2. The NP positions acquired at low pressures ($p$ < 1 mBar) are filtered by a frequency domain bandpass filter (passband for the $x$ axis: 87–96 kHz, passband for the $y$ axis: 72–90 kHz) in order to keep only the leading oscillation in the selected axis because at low pressures crosstalks between different axes become visible in PSD and influence the analyses of particle motion in a selected axis.
3. Using the filtered NP positions $x\left( t \right)$ determined in equidistant time steps ${\Delta }t$, we calculate NP velocities $v_{x} \left( t \right)$ using the central difference rule $v_{x} \left( t \right) = \left[ {x\left( {t + \Delta t} \right) - x\left( {t - \Delta t} \right)} \right]/2\Delta t$, where $\Delta t$ is given by the sampling frequency during the data acquisition $\Delta t = 1/f_{{{\text{sample}}}} = 1/1.78$ μs. Furthermore, we verified by the computer simulations that the experimental sampling frequency is sufficient to calculate the instantaneous particle velocity even at atmospheric pressure.
4. We look for an event when both position and velocity pass through a small region of the phase space $x_{0} - \Delta x_{0} \le x_{0} \le x_{0} + \Delta x_{0}$ and $v_{x0} - \Delta v_{x0} \le v_{x0} \le v_{x0} + \Delta v_{x0}$, where both $\Delta x_{0}$ and $\Delta v_{x0}$ are small but slightly bigger than the experimental uncertainty of measured positions and calculated velocities. If we find several consecutive points in this region, the selected one gives the minimal separation from $x_{0}$. The uncertainties were estimated by the high frequency tail of the PSD at frequencies above 660 kHz (see Calibration of quadrant photodetector in Methods) and they correspond to the white noise having standard deviation $\sim 2$ nm, which is about 3% of the particle standard deviation from the optical trap center.
5. When such an event is detected, we assume it is the beginning of a new trajectory corresponding to $t = 0$ and we take a snippet of an on-going trajectory of length $\delta t$ (typically $\delta t \simeq 100$ μs). This snippet is added into a statistical ensemble of trajectories starting at the vicinity of the point $\left( {x_{0} ,v_{x0} } \right)$ in the phase space.
6. Steps (4) and (5) are applied on the remaining part of the acquired NP record while over-lapping parts of the trajectories are not included.
7. Averaged trajectory $\langle x\left( t \right) \rangle$, velocity $\langle v_{x} \left( t \right) \rangle$ and their variances ${\text{Var}} \left( x \right) = \langle x^{2} \left( t \right) \rangle - \langle x\left( t \right )\rangle^{2}$, ${\text{Var}} \left( {v_{x} } \right) = \langle v_{x}^{2} \left( t \right) \rangle- \langle v_{x} \left( t \right)\rangle^{2}$ are calculated from the statistical ensemble obtained in the previous steps. Each ensemble may contain from tens up to $\sim 5 \times 10^{4}$ snippets of trajectories depending on the selected initial conditions.

Several examples showing such averaged trajectories starting at points $\left( {x_{0} ,0} \right)$ are shown in Fig. 2f where the damping of oscillator amplitudes can be clearly seen as well as the frequency dependence on the oscillator amplitude. Furthermore, Fig. 2g shows the increase in the position variance ${\text{Var}}\left( x \right)$ determined for the particular initial condition $\left( {x_{0} ,v_{x0} } \right) = \left( {0,{ }0} \right) \pm \left( {2\,{\text{nm}},\;1\,{\text{mm}}\,{\text{s}}^{{ - {1}}} } \right)$. After a short transient process the variance achieves a saturated value which corresponds to the thermalization of the particle motion to the surrounding bath.

## Methods for determination of parameters of the Duffing oscillator

Below we present a description of four methods we use for estimation of the parameters of the nonlinear Duffing-type oscillator. At the end of each subsection describing one method the procedure for the parameter determination is summarized.

### The Duffing oscillator approximation (DOA)

In many types of nonlinear oscillators with a harmonic potential minimum, a nonlinearity only weakly modifies harmonic oscillations. In this case it is sufficient to add only the first nonlinear term to the harmonic potential profile description given by a Taylor series expansion^45, 55^. On time scales shorter than any thermalization, the motion of the so-called Duffing oscillator can be described by the deterministic Duffing equation (DDE) in the following form

2 $$\ddot{x} + \Gamma \dot{x} + \Omega_{0}^{2} x\left( {1 - \xi x^{2} } \right) = 0,$$

where $x$ denotes the particle position along one axis, ${\Gamma } = \gamma /m$ denotes the medium damping with $m$ and $\gamma$ being the mass of the oscillator (e.g. a levitating NP) and drag coefficient of the medium (e.g. air at low pressure), respectively. ${\Omega }_{0} = \sqrt {{\upkappa }/m}$ denotes the eigenfrequency of the ideal harmonic oscillator with the oscillator stiffness $\kappa$ (e.g. the stiffness of the optical trap). $\xi$ represents the coefficient of Duffing nonlinearity and is related to the third order coefficient of the Taylor expansion of the optical force near the potential minimum at $x = 0$. The softening Duffing oscillator described in this way will be subject of our following analyses.

Local analysis near $x = 0$ of such Duffing oscillator for weak damping ${\Gamma }$ leads to a solution of the lowest perturbation order in the form of a damped oscillator^56^

3 $$x\left( t \right) = x_{0} \exp \left( { - \frac{1}{2}\Gamma t} \right)\cos \left( {\Omega_{{\text{D}}} t + \theta_{0} } \right),$$

where $x_{0}$ and $\theta_{0}$ are given by the initial conditions at $t = 0$. The frequency $\Omega_{{\text{D}}}$ can be expressed as^56^

4 $$\Omega_{{\text{D}}}^{2} = \Omega_{0}^{2} \left( {1 - \frac{3}{4}\xi x_{0}^{2} } \right) - \left( {\frac{\Gamma }{2}} \right)^{2} .$$

Considering only a weakly damped nonlinear oscillator we can define the eigenfrequency of the damped harmonic oscillator $\Omega_{{\text{H}}}$ depending on its initial amplitude as

5 $$\Omega_{{\text{H}}} = \sqrt {\Omega_{{\text{D}}}^{2} + \left( {\Gamma /2} \right)^{2} }$$

6 $$\simeq \Omega_{0} \left( {1 - \frac{3}{8}\xi x_{0}^{2} } \right).$$

In reality, the micro- or nano-oscillators are driven by thermal noise which is generally assumed as the white noise and the DDE modifies to the Langevin type of stochastic Duffing equation (SDE) in the following form^18, 45^

7 $$\ddot{x} + \Gamma \dot{x} + \Omega_{0}^{2} x\left( {1 - \xi x^{2} } \right) = \frac{{F^{{{\text{fluct}}}} }}{m},$$

where the broadband fluctuating Langevin force $F^{{{\text{fluct}}}}$ is uncorrelated in time with zero mean and its variance is given by the fluctuation–dissipation theorem $\left\langle {F^{{{\text{fluct}}}} \left( t \right)} \right\rangle = 0,\left\langle {{ }F^{{{\text{fluct}}}} \left( t \right)F^{{{\text{fluct}}}} \left( {t^{\prime}} \right)} \right\rangle = 2m{\Gamma }k_{{\text{B}}} T\delta \left( {t - t^{\prime}} \right)$, where $\left\langle \ldots \right\rangle$ denotes correlation of functions within brackets and $\delta \left( x \right)$ denotes the delta function. This equation is later used for numerical simulations of the dynamics, results are presented in Supplementary Information. Even though Eq. (7) assumes the driving force with a white noise spectrum, the simulations and experimental activities can be extended to a driving force with a coloured noise spectrum, e.g. using an external force with well-controlled frequency spectrum.

#### Idealized harmonic oscillations

For initial conditions close to the potential minimum, the weakly damped nonlinear oscillator behaves predominantly as harmonic^18^ and a transient motion can be well described by a harmonic oscillator with an eigenfrequency $\Omega_{0}$ continuously damped with rate $\Gamma$ and excited by thermal surrounding characterized by the effective transient temperature $T_{{{\text{TR}}}}$. It is sufficient to select initial conditions at the potential minimum with zero speed and observe heating and random build-up of many linearized small oscillations. We use the post-selection process described in the previous section, in order to virtually cool down the NP and afterwards the NP motion under the influence of the heat transfer from surroundings is analyzed. This is equivalent to a direct observation of the particle heating in experimental systems^57^. If we select the initial conditions at $\left( {x_{0} ,v_{x0} } \right) = \left( {0,{ }0} \right) \pm \left( {2\,{\text{nm}},1\,{\text{mm}}\,{\text{s}}^{ - 1} } \right)$ and determine ${\text{Var}}\left( x \right)$ from the experiment for long enough acquisition time to cover the thermalization, we can fit the variance profile very well using the analytical solution of the linearized harmonic oscillator^24^

8 $${\text{Var}}\left( x \right) = \frac{{k_{{\text{B}}} T_{{{\text{TR}}}} }}{{m\Omega_{0}^{2} }}\left\{ {1 - {\text{e}}^{{ - \Gamma {\text{t}}}} \left[ {\frac{{\Omega_{0}^{2} }}{{\Omega_{{\text{D}}}^{2} }} - \frac{\Gamma }{{2\Omega_{{\text{D}}} }}\sin \left( {2{\Omega }_{{\text{D}}} t} \right) + \frac{{\Gamma^{2} }}{{4\Omega_{{\text{D}}}^{2} }}\cos \left( {2\Omega_{{\text{D}}} t} \right)} \right]} \right\}.$$

**Figure 3:**
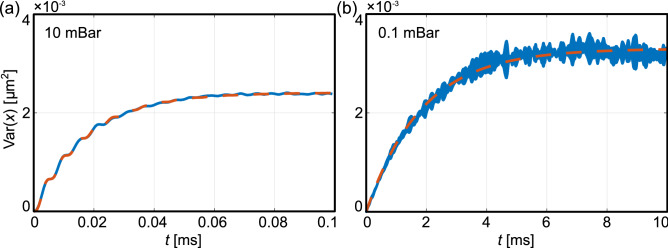
Time dependent increase in variance ${\text{Var}}\left( x \right)$ of the ensemble of trajectories with initial conditions close to $\left( {x_{0} ,v_{x0} } \right) = \left( {0, 0} \right)$ for different pressures. Blue curves depict experimental data and red dashed curves are fits (**a**) by Eq. (8) and (**b**) by Eq. (9). The values of fitted parameters are ${\Omega }_{0} /\left( {2\pi } \right) = \left( {92 \pm 2} \right)$ kHz, ${\Gamma } = \left( {58.0 \pm 0.5} \right) \times 10^{3}$ s^−1^, $T = \left( {24 \pm 1} \right)$ °C; and ${\Gamma } = \left( {467 \pm 2} \right)$ s^−1^, $T = \left( {125.5 \pm 0.5} \right)$ °C for pressures $p = 10$ and $p = 0.1$ mBar, respectively.

From the fit to the experimental data (see Fig. 3a) we can determine all three parameters $\Omega_{0}$, $\Gamma$ and $T_{{{\text{TR}}}}$. These parameters remain constant even if the NP enters the nonlinear region. As a cross-check, we can use the variance of velocities, which provides us with the same results. In a weakly damped system with $\Gamma \ll \Omega_{{\text{D}}}$, we can use a simplified formula without oscillations in order to quantify the heating of the system, i.e. medium damping as well as the effective temperature using

9 $${\text{Var}}\left( x \right) = \frac{{k_{{\text{B}}} T_{{{\text{TR}}}} }}{{m\Omega_{0}^{2} }}\left( {1 - {\text{e}}^{{ - \Gamma {\text{t}}}} } \right).$$

Such fit to the experimental data for a weakly damped oscillator is shown in Fig. 3b.

#### A few nonlinear oscillations

A nonlinearity in the potential profile influences the NP motion if an NP initial position is placed far from the potential minimum. If the damping is weak, i.e. $\Gamma \ll \Omega_{0}$, and initial oscillator amplitude is sufficiently large, the NP amplitude does not change significantly over a few periods neither due to the damping nor due to the thermal heating (see Fig. 2f). In this regime, the averaged trajectories or phase space portraits follow very well deterministic nonlinear damped oscillations with negligible stochastic driving term, as described by Eqs. (2–6).

**Figure 4:**
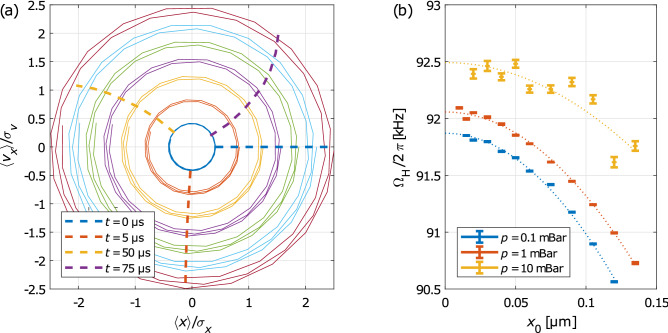
(**a**) Phase portraits of the NP motion reconstructed using the averaged experimental data normalized to standard deviations. The phase space trajectories drawn over 50 µs starting at different initial positions $x_{0}$ = 20, 40, 60, 75, 90, 105, and 120 nm, fixed initial velocity $v_{x0} = 0$ and ambient pressure 1 mBar. The dashed curves connect the phase space positions corresponding to the same times $t$ but different initial positions. Normalizing factors $\sigma_{x}$ and $\sigma_{v}$ denote $\sqrt {{\text{Var}}\left( x \right)}$ and $\sqrt {{\text{Var}}\left( {v_{x} } \right)}$, respectively. (**b**) Eigenfrequency of a damped Duffing oscillator ${\Omega }_{{\text{H}}}$ as a function of the initial NP amplitude $x_{0}$ for various ambient pressures is obtained from parameter fitting by Eq. (3) to the data similar to Fig. 2f. The fit by Eq. (6) is plotted by dotted curves.

Figure 4a shows an example of phase portraits obtained for different initial conditions $\left( {x_{0} ,0} \right)$ at the short time scale (< 1 ms) of such the regime. Due to the low damping, trajectories spiral slowly inwards towards the equilibrium point and stay on their orbits. The dashed curves, resembling clock hands, show the NP position in the phase space for different oscillation amplitudes and at given times from the initial condition denoted as the blue dashed straight line. As the oscillation evolves, the dashed curves bend backwards indicating that the outer phase space trajectories orbit with a lower frequency. Figure 4b confirms the dependence of ${\Omega }_{{\text{H}}}$ on the initial conditions $\left( {x_{0} ,v_{x0} } \right)$ and shows the expected parabolic profile described by Eq. (6).

*Determination of* ${\Gamma}$: Fitting Eq. (9) to the variance ${\text{Var}}\left( x \right)$ of trajectory ensembles starting at $\left( {x_{0} ,v_{x0} } \right) = \left( {0,0} \right) \pm \left( {2\,{\text{nm}},1\,{\text{mm}}\,{\text{s}}^{{ - {1}}} } \right)$ we obtained the damping coefficient ${\Gamma }$ and the saturated-level pre-factor $k_{{\text{B}}} T/\left( {m{\Omega }_{0}^{2} } \right)$ (see Fig. 3). Alternatively for higher ambient pressures we obtained the damping coefficient ${\Gamma }$, eigenfrequency ${\Omega }_{0}$ and bath temperature $T$ by fitting Eq. (8) to the same dataset.

*Determination of* ${\Omega}_{{\text{D}}}$: Fitting Eq. (3) to the mean position of trajectory ensembles $x\left( t \right)$ over a few periods ($\sim 10$) we determined the oscillation frequency ${\Omega }_{{\text{D}}}$ (see Fig. 2f). The initial amplitudes $x_{0}$ may reach up to $\sim 2 \times$ the standard deviation of particle positions in the trap. For even bigger initial displacements the size of trajectory ensemble becomes very small and the results of analysis are unreliable.

*Determination of* ${\Omega }_{{0}}$* and *$\xi$: Employing Eq. (5) with ${\Omega }_{{\text{D}}}$ and ${\Gamma }$ determined above gives the eigenfrequency of a damped Duffing oscillator ${\Omega }_{{\text{H}}}$. Fitting parabolic dependence of Eq. (6) to ${\Omega }_{{\text{H}}}$ for different initial conditions $x_{0}$ gives the eigenfrequency ${\Omega }_{{0}}$ and the coefficient of Duffing nonlinearity $\xi$ (see Fig. 4b).

*Determination of* $T$: Determination of ${\Gamma }$ in the first step above gave the pre-factor $k_{{\text{B}}} T/\left( {m{\Omega }_{0}^{2} } \right)$ or directly the effective temperature $T$. Since ${\Omega }_{{0}}$ is known from the previous step, the pre-factor gives the effective temperature $T$ (knowing the NP diameter 170 nm and its density $2000\,{\text{kg/m}}^{3}$).

### Numerical solution of deterministic Duffing equation (DDE)

To check the accuracy of the approximative solutions given by Eqs. (3–6), we solve DDE given by Eq. (2) using the direct numeric integration (Matlab, ODE45 function) under the selected initial conditions. We use this procedure to search for parameters (${\Gamma }$, ${\Omega }_{0}$, and $\xi$ ) giving the best coincidence between the numerical solution of DDE and experimental data corresponding to a few nonlinear oscillations presented in Sect. 1 (see also an example in Fig. 2f).

*Determination of *${\Gamma }$*, *${\Omega }_{0}$*, and *$\xi$: We fitted numerical solution of Eq. (2) to all averaged trajectories $x\left( t \right)$ (for various initial conditions $\left( {x_{0} ,0} \right)$) at once. The contribution of trajectories was weighted as $x_{0}^{ - 2}$ so that all trajectories contributed to the residual sum with the same weight.

### Power spectral density (PSD)

For comparison with other methods we also used this classical method. PSD does not depend on initial conditions, therefore, it is not proper to characterize the transient dynamics. It can be used only to observe similarities and difference between the oscillator parameters at the short-time and long-time scale. Furthermore, this method does not allow to determine the coefficient of nonlinearity because only the harmonic potential is assumed to get Eq. (1).

*Determination of* $\Gamma$* and* ${\Omega}_{0}$: Fitting Eq. (1) to experimental ${P}_{xx}$ gives ${\Omega }_{0}$ and $\Gamma$.

*Determination of T* :  In the case that the calibration constant of the position detector is determined by other means we can use the velocity PSD ${P}_{vv}$, see Eq. (11) and Methods, in order to determine the spectral effective temperature. In such a case the spectral temperature can be expressed as^29^

10 $${T}_{\text{SP}}=\frac{m}{\pi {k}_{\text{B}}}{\int }_{0}^{\infty }{P}_{vv}\left(\omega \right){\text{d}}\omega .$$

For this approach the limiting factor is the determination of high frequency shot noise, see Methods, or the actual limits of the integral achievable from the experiment.

### Numerical solution of stochastic Duffing equation (SDE)

In order to verify the described methods used for data processing and determination of parameters of the Duffing oscillator, we simulate the random particle motion in 1D described by the SDE given by Eq. (7) based on the Verlet scheme^58^. This approach is also generalized to 3D and corrected for influences of the other axes, as we described in more details in Supplementary Information.

We simulated 200 trajectories of an NP with random initial conditions with a time step corresponding to the experimental sampling frequency (total duration of a single trajectory was 1 s). The data were processed in the same way as experimental data and the obtained parameters were compared with the input ones.

## Results and discussion

**Figure 5:**
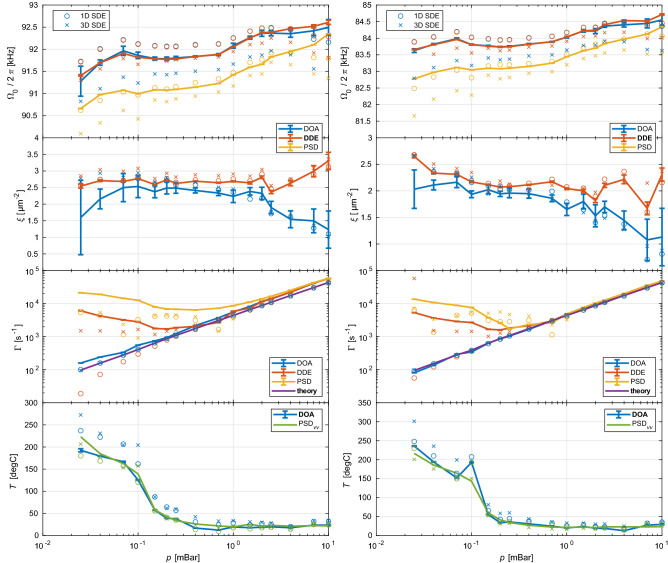
Comparison of pressure dependence of parameters of the Duffing oscillator represented by the optically levitated particle in a nonlinear optical potential. Left and right columns correspond to an NP oscillating along the $x$ and $y$ coordinate axis, respectively. (row 1) Eigenfrequency ${\Omega }_{0}$, (row 2) the parameter of Duffing nonlinearity $\xi$ , (row 3) the damping factor ${\Gamma }$, (row 4) effective temperature $T$, (all panels) full curves: DOA (blue), DDE (red), PSD (orange). Circles $\circ$ and crosses $\times$ correspond to 1D and 3D simulations from SDE analysed by different methods, where we used parameters obtained by the analysis of experimental data with the method marked bold in the legend, for more information see Supplementary Information. Colour of each symbol corresponds to the method of analysis depicted in the legend. All errorbars correspond to the 95% confidence intervals of the given quantity and are either directly based on the results of the nonlinear least square fitting or the results of the fits are combined by the error propagation law that gives the depicted intervals, respectively.

We have acquired data at several levels of ambient pressure, processed in a way described in the previous section for different initial conditions and determined oscillator’s parameters using methods DOA, DDE and PSD. Further, we obtained trajectories using SDE by means of computer simulations for parameters determined from the experiment and processed in the same way as the measured trajectories, see also Supplementary Information. Figure 5 compares the obtained parameters for the $x$ (left) and $y$ (right) axes. Other possible methods how to determine parameters of the Duffing oscillators are summarized in Methods. Simulations for huge set of input parameters are analyzed in Supplementary Information and verify applicability and possible bias of each method.

### Eigenfrequency ${{\varvec{\Omega}}}_{0}$

${\Omega }_{0}$ obtained by DOA and DDE methods are about 1 kHz higher comparing to the PSD method. This is caused by the fact that ${\Omega }_{0}$ for the Duffing oscillator corresponds to the extrapolated oscillation frequency with zero amplitude $x_{0}$ which is the highest frequency, see Eq. (6). In contrast, the PSD method averages oscillation frequencies of all amplitudes.

Only for the highest pressure (10 mBar) values of ${\Omega }_{0}$ obtained by all methods are in agreement. In this case the dominant effect in the PSD method is the peak broadening by the damping and the resonant peak is not influenced by the nonlinear behaviour at all. The frequency drift through different pressures (same in both axes) is caused by the fluctuation of the total optical power in the trapping laser beam.

The analysis of the 1D simulated data follows the same trends as in the case of experimental data. In case of DDE method a weak bias $\sim$ 0.3 % towards higher frequencies appears. On the contrary the analysis of 3D simulations shows that values of eigenfrequency ${\Omega }_{0}$ obtained by all methods are shifted by a similar value towards lower frequencies, involving corrections described in Supplementary Information. Similar results were also confirmed by the analysis of SDE simulated data with various input conditions (see Supplementary Information). Therefore, we are persuaded the DOA and DDE methods provide valid ${\Omega }_{0}$ for weakly nonlinear systems of optically levitated particles.

### The coefficient of Duffing nonlinearity ${\varvec{\xi}}$

DDE method gives slightly higher values of coefficient of nonlinearity than DOA with the mean value and standard error in the $x$ and $y$ axes equal to $\xi_{x} = \left( {2.7 \pm 0.2} \right)\,{\upmu}{\text{m}}^{ - 2}$ and $\xi_{y} = \left( {2.1 \pm 0.2} \right)\,{\upmu}{\text{m}}^{ - 2}$, respectively. Discrepancy between both methods increases for ambient pressure above 2 mBar where values from DOA method drop down significantly. At these pressures the oscillations are strongly damped and the eigenfrequency ${\Omega }_{{\text{H}}}$ can not be determined with sufficient precision (see the yellow curve in Fig. 4b) where the fit by Eq. (6) fails to give reliable results.

If the DOA and DDE methods were applied on the simulated data (both 1D and 3D), the DDE method returns values corresponding to the input values of the simulations. The DOA method gives the same values as DDE but deviates in the same way as experimental data for higher pressures.

The values obtained with DOA and DDE can be compared with a theoretical estimate of $\xi$ for the Gaussian beam with the beam waist $w_{0}$ under Rayleigh approximation^18, 44^ using $\xi = 2/w_{0}^{2}$ that gives $\xi = 2.76\,{\upmu}{\text{m}}^{-2}$. The beam waist in the focal plane $w_{0} = 0.85\,{\upmu}{\text{m}}$ was determined from Zemax software package in accordance to the aberrations of the focusing lens at the trapping wavelength but ignoring polarization effects in such non-paraxial beam. Thus the obtained beam waist is the same for the $x$ and $y$ axes and approximately two times wider comparing to the direct utilization of numerical aperture of ideal focusing optics. Moreover, the particle diameter of 170 nm is slightly bigger than Rayleigh approximation allows, therefore the force profiles deviate from the expected simple gradient of optical intensity dependence which may also slightly influence the value of the coefficient of nonlinearity. Consequently the value of $\xi$ predicted from the Gaussian beam waist corresponds quite well with the values determined from the experimental trajectories.

### The damping coefficient ${{\varvec{\Gamma}}}$

Values of medium damping ${\Gamma }$ obtained with DOA, DDE and PSD for different pressures from the experimental data are also compared with the theoretical model^47^, where we assumed nitrogen molecule diameter 0.372 nm and viscosity 17.7 µPa s. Only the DOA method gives a very good coincidence with the theoretical model in the whole range of investigated pressures. DDE and PSD methods follow well the theory for pressures above 0.5 mBar however for lower pressures ${\Gamma }$ values obtained by those two methods differ from the theoretical predictions by one order or even more. Since the peak in experimental PSD broadens and gets asymmetric at lower pressures due to the nonlinearity in the system (see Fig. 2d,e), its fit by a function derived for an ideal harmonic oscillator results in a larger ${\Gamma }$. In the case of 1D SDE simulated data the DDE method follows well the theoretical predictions (see red circles in row 3 of Fig. 5) while the analysis of 3D simulated trajectories by the DDE method (red crosses) reveals the same trend as the analysis of experimental data. On the contrary the DOA analysis of variance increase in the experimental data as well as in 1D or 3D SDE simulated trajectories leads to the almost perfect coincidence with the values predicted by the theoretical model. Thus we conclude that the increased damping obtained by DDE method is caused by the crosstalks between coordinate axes (especially between lateral and longitudinal ones).

### Effective temperature ${\varvec{T}}$

The last parameter describing dynamics of levitated NP in a Duffing type potential is the effective temperature *T*. We compared the temperature obtained by the position variance in DOA method (referred to as transient temperature *T*_TR_) and by the integration of the area under the oscillation peak in velocity PSD (PSD*vv*), referred to as the spectral temperature *T*_SP_^29^. We note we fixed the value of the calibration constant obtained at pressures higher than 0.4 mBar, as we explain in Methods. Both methods provide us with comparable results due to the fact that the energy of particle motion contained in PSD*vv* is independent of the nonlinear effects. We see that $T$ is constant for pressures above 0.5 mBar and corresponds to the ambient temperature. For lower pressures the effective temperature firstly slowly increases which is followed by the steep increase for even lower pressures up to $\sim$ 500 K. This behaviour corresponds to the temperature increase observed in^57^ and was explained by a decrease of the heat dissipation by conduction at low pressures. Unfortunately, our system does not allow us to reach even lower pressures ($< 10^{ - 2}$ mBar) where one would expect the saturation of temperature since all absorbed heat would be dissipated only by the radiation independent of ambient pressure. The increase in the temperature is evident from the saturation level of the position variance shown in Fig. 3. For a higher pressure of 10 mBar (Fig. 3a) the saturation level is lower compared to the variance at pressure of 0.1 mBar shown in Fig. 3b. The temperature obtained by the analysis of the simulated trajectories corresponds well to the experimental values that were used as simulation inputs.

## Conclusions

Parameters of a nonlinear oscillator are usually estimated from steady states but the oscillator parameters found during the transient dynamics can be significantly different. The ability to thoroughly characterize the nonlinear oscillator on the short time scale is crucial for experimental testing of transient dynamics of levitating particles used in nanosensing and thermodynamical engines. The parameters are predicted even before the heating rate considerably affects the experiment which is desirable e.g. for quantum experiments with a prepared initial state. We introduced new transient methods to characterize parameters of an optically levitated NP behaving as a weakly nonlinear Duffing oscillator. The novel approach is based on post-processing of the acquired experimental data in such a way that for each selected initial state in the phase space, an ensemble of short evolutions is collected. From mean position and momentum and their variances, a Duffing oscillator can be fully characterized through its eigenfrequency, damping, coefficient of nonlinearity or temperature. The described methodology can be used also for very low temperatures down to quantum mechanical motion.

We developed two methods, i.e. Duffing oscillator approximation (DOA) and deterministic Duffing equation (DDE), and we applied them on experimental data and on datasets obtained from stochastic Duffing equation (SDE) for a wider range of experimentally accessible parameters. The comparisons between the methods and with the widely used steady-state method of power spectral density (PSD) assuming only an ideal harmonic oscillator are revealed in Fig. 5 and discussed in detail in the previous section. The comparison with SDE verified the reliability of parameters extracted by the methods for one-dimensional and three-dimensional motion of the NP. Focusing only on the parametric region corresponding to the experimental parameters we conclude that both DOA and DDE determine eigenfrequency ${\Omega }_{0}$ with a precision better than 1 % and nonlinearity $\xi$ with a precision better than 14 % for pressures lower than 1 mBar. DOA gives damping factor $\Gamma$ with a precision better than 1 % for all considered pressures, nonlinearities and temperatures. In contrast velocity PSD method gives temperature estimate within 1 % while DOA within 14 % for pressures between 0.01 and 10 mBar.

Moreover, the trajectories simulated by the SDE could be in principle used directly to obtain the parameters of the experimental system together with the concept of machine learning^59^. Here the artificial neural network (ANN) would be trained using the simulated trajectories or some features acquired of such trajectories and later the ANN would predict parameters of the experimental system based on the measured data. Yet this approach is extremely time consuming and computationally demanding and contradicts our approach in this paper to develop a simple and fast method to characterize oscillator parameters and optical trap properties. In conclusion, the presented transient methods can reliably characterize all important parameters describing the Duffing oscillator especially for lower pressures (under 1 mBar), where the nonlinearity plays a significant role and a peak profile in PSD starts to deviate from the ideal Lorentzian shape.

## Methods

### Calibration of quadrant photodetector

For the position calibration of the quadrant detector we used a method based on an integrating of the velocity PSD $P_{vv}$^29^ which was calculated directly from the position PSD $P_{xx}$ as

11 $$P_{vv} = \left( {P_{xx} - P_{xx}^{\infty } } \right)\omega^{2} .$$

$P_{xx}^{\infty }$ is the average level of the white (shot) noise at high frequencies in $P_{xx}$. The calibration constant is then calculated as

12 $$C_{{{\text{V}} \to {\text{m}}}} = \sqrt {\frac{{\pi k_{{\text{B}}} T}}{{m\mathop \smallint \nolimits_{0}^{\infty } P_{vv} {\text{d}}\omega }}} ,$$

where $T = 295$ K is the room temperature. To determine the noise level $P_{xx}^{\infty }$ we analyzed $P_{xx}$ for frequencies $\omega /2\pi > 0.75f_{{{\text{Nyq}}}}$, where $f_{{{\text{Nyq}}}} = f_{{{\text{sample}}}} /2$ is the Nyquist frequency and the sampling frequency $f_{{{\text{sample}}}} = 1.78$ MHz. In this range the PSD was firstly smoothed by a moving average filter and then the result was fitted by

13 $$P_{xx} \left( f \right) = Af^{ - B} + C,$$

where $A$, $B$, and $C$ are fitted parameters. If $B > 0$ we set the noise level as $P_{xx}^{\infty } \equiv C$, otherwise we set the noise level to the mean value of the high frequency part ($\omega /2\pi >$ 800 kHz) of $P_{xx}$.

**Figure 6:**
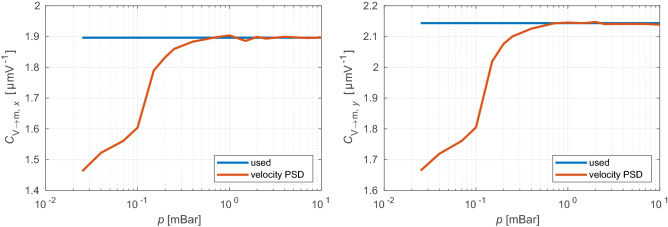
Pressure dependence of calibration constant $C_{{{\text{V}} \to {\text{m}}}}$ calculated from the PSD $P_{vv}$ (red) and a value of calibration constant that was actually used for the data processing (blue) for the $x$ (left) and $y$ (right) axes.

The recovered calibration factor $C_{{{\text{V}} \to {\text{m}}}}$ forms a constant level plateau for pressures above 0.4 mBar (see Fig. 6) which is consistent with the previously published results^29^. For lower pressures below 0.4 mBar the calibration constant $C_{{{\text{V}} \to {\text{m}}}}$ starts to drop. This can be attributed to the change of the NP temperature leading to a higher temperature of the NP center of mass motion than the room temperature used for calculation of the red curves in Fig. 6. Therefore, we applied the value of calibration constant equal to mean value of $C_{{{\text{V}} \to {\text{m}}}}$ in the pressure range from 0.4 mBar up to 10 mBar to all studied pressures.

### List of all possible methods for determination of parameters of the Duffing oscillator

In the following section we present an overview of several alternative ways that can be used for determination of parameters describing a Duffing oscillator. The mentioned methods are explained in more details in the main text.

#### Determination of ${\Gamma }$

1. Fitting Eq. (9) to the variance of trajectory ensembles ${\text{ Var}}\left( x \right)$ the damping coefficient ${\Gamma }$ and the saturated-level pre-factor $k_{{\text{B}}} T/\left( {m{\Omega }_{0}^{2} } \right)$ are obtained.
2. Fitting Eq. (8) to the variance of trajectory ensembles ${\text{Var}}\left( x \right)$ for higher ambient pressure, the damping coefficient ${\Gamma }$, eigenfrequency ${\Omega }_{0}$ and effective temperature $T$ are obtained.
3. Fitting Eq. (3) to the mean position of trajectory ensembles $x\left( t \right)$ over a few periods, medium damping ${\Gamma }$ and the oscillation frequency ${\Omega }_{{\text{D}}}$ are determined.
4. Fitting Eq. (1) to the position PSD gives ${\Gamma }$ assuming an ideal harmonic oscillator.
5. Fitting Eq. (11) to the velocity PSD*vv* gives ${\Gamma }$ assuming an ideal harmonic oscillator.
6. Fitting numerical solution of Eq. (2) to all averaged trajectories $x\left( t \right)$ (for various initial conditions $\left( {x_{0} ,0} \right)$) at once gives values of ${\Gamma }$, ${\Omega }_{0}$ and $\xi$.

#### Determination of ${\Omega }_{{\text{D}}}$

7. Step (3) gives also the oscillation frequency ${\Omega }_{{\text{D}}}.$

#### Determination of ${\Omega }_{{\text{0}}}$

8. Employing Eq. (5) with ${\Omega }_{{\text{D}}}$ and ${\Gamma }$ determined above gives the eigenfrequency of a damped Duffing oscillator ${\Omega }_{{\text{H}}}$. Fitting parabolic dependence of Eq. (6) to ${\Omega }_{{\text{H}}}$ for different initial conditions $x_{0}$ gives the eigenfrequency ${\Omega }_{0}$ and the coefficient of Duffing nonlinearity $\xi .$
9. Step (2) gives also ${\Omega }_{0}$.
10. Step (4) gives also ${\Omega }_{0}$.
11. Step (5) gives also ${\Omega }_{0}$.
12. Step (6) gives also ${\Omega }_{0}$.
13. In the case of three-dimensional nanoparticle motion, one should use the frequency correction to get the eigenfrequency comparable to one-dimensional case, see Supplementary Information.

#### Determination of $\upxi$

14. Step (8) gives also coefficient of Duffing nonlinearity.
15. Step (6) gives also.

#### Determination of T

16. Step (1) gives the pre-factor ${k}_{\text{B}}T/\left(m{\Omega }_{0}^{2}\right)$. With the known ${\Omega }_{0}$ and oscillator mass, $T$ can be calculated.
17. In the case that the position detector is already calibrated, the velocity PSD can be used to determine the spectral temperature, employing Eq. (10).

## Supplementary information

Supplementary Information

## Data Availability

The data that support the plots within this paper and other findings of this study are available from the corresponding author upon reasonable request.
