# Supplementary material for: Using the transient trajectories of an optically levitated nanoparticle to characterize a stochastic Duffing oscillator
Source: Sci Rep. 2020 Sep 2;10:14436. doi: 10.1038/s41598-020-70908-z (PMC7468157; doi:10.1038/s41598-020-70908-z)
Supplement: Supplementary file 1 — Supplementary Information [file 41598_2020_70908_MOESM1_ESM.pdf]

Supplementary Information: Using the transient trajectories of an optically levitated nanoparticle to characterize a stochastic Duffing oscillator

Jana Flajšmanová[1], Martin Šiler[1,*], Petr Jedlička[1], František Hrubý[1], Oto Brzobohatý[1], Radim Filip[2], and Pavel Zemánek[1,+]

[1]Institute of Scientific Instruments of the Czech Academy of Sciences, Královopolská 147, 612 64 Brno, Czech Republic
[2]Department of Optics, Palacký University, 17. listopadu 1192/12, 771 46 Olomouc, Czech Republic
[*]siler@isibrno.cz
[+]zemanek@isibrno.cz

1 1D and 3D stochastic simulations

We consider that the NP is localized in a laser beam with a Gaussian profile of the beam intensity in all three axes. Further, let us assume that even though there is the scattering force pushing the NP in the positive direction of the z axis, this contribution is negligible and we will consider a motion of the NP influenced by the so–called gradient forces only. The neglected scattering force just slightly shifts the NP equilibrium position along the z axis beyond the beam focus.

In the 1D case the numerical solution of stochastic Duffing equation [1, 2] is obtained using the random particle motion based on the Verlet scheme [3]. However in the experiment, the NP moves in 3D potential and thus we extended this equation to three axes by extending the force term. Let us assume the 3D conservative potential follows the intensity shape of the Gaussian beam in all three axes:

$$U(\mathbf{r}) = -U_0\, \mathrm{e}^{-2\frac{x^2}{{w_x}^2}-2\frac{y^2}{{w_y}^2}-2\frac{z^2}{{w_z}^2}}, \tag{S1}$$

where U_0 is the depth of the trapping potential which is related to the total laser power, w_x, w_y, and w_z are the radii of the Gaussian beam waists along the coordinate axes, respectively. Assuming conservative forces only we may express the force acting on the NP as $\mathbf{F} = -\nabla U(\mathbf{r})$ and we get all individual force components:

$$F_x = -2\xi_x\, U_0\, x\, \exp\left[-\xi_x x^2 - \xi_y y^2 - \xi_z z^2\right], \tag{S2}$$
$$F_y = -2\xi_y\, U_0\, y\, \exp\left[-\xi_x x^2 - \xi_y y^2 - \xi_z z^2\right], \tag{S3}$$
$$F_z = -2\xi_z\, U_0\, z\, \exp\left[-\xi_x x^2 - \xi_y y^2 - \xi_z z^2\right], \tag{S4}$$

where we used the relation between the beam waist and the coefficient of nonlinearity [1, 4]

$$\xi_i = 2/w_i^2, \tag{S5}$$

where $i = x, y, z$. Further, we express the forces using the eigenfrequency $\Omega_{0i} = \sqrt{2\xi_i U_0/m}$ corresponding to axis i and simplify the optical force expression using a Taylor expansion for coupled Duffing oscillators:

$$F_x = -m\Omega_{0x}^2\, x\, \exp(-\Psi) \simeq -m\Omega_{0x}^2\, x\, (1-\Psi), \tag{S6}$$
$$F_y = -m\Omega_{0y}^2\, y\, \exp(-\Psi) \simeq -m\Omega_{0y}^2\, y\, (1-\Psi), \tag{S7}$$
$$F_z = -m\Omega_{0z}^2\, z\, \exp(-\Psi) \simeq -m\Omega_{0z}^2\, z\, (1-\Psi), \tag{S8}$$
$$\Psi = \xi_x\left(x^2 + \frac{\Omega_{0y}^2}{\Omega_{0x}^2}y^2 + \frac{\Omega_{0z}^2}{\Omega_{0x}^2}z^2\right). \tag{S9}$$

We simulated 200 trajectories of the NP for each combination of parameters with random initial conditions with a time step corresponding to the experimental sampling frequency $f_{\mathrm{sample}} = 1.78$ MHz (total duration of a single trajectory was 1 s).

The values of parameters characterizing a Duffing oscillator used for simulations were taken from the experimental results obtained by methods that proved to be the most reliable. In case of 1D simulations, parameters Ω_0 and ξ were given by experimental results obtained by DDE method, DOA method was used for T values and Γ was calculated by the theoretical model [5]. The simulated trajectories were further evaluated by all the methods described in the manuscript and the results of such processing are plotted in Fig. 5 in the main text as the circles. Let us remark, the parameters of the oscillator obtained from the 1D simulations coincide with those obtained from the experimental records. The only exception is Γ obtained by the DDE method which follows the input theoretical values even for low pressure region of Fig. 5 in the main text.

In the case of full 3D simulations we took the values of Ω_{0x} and Ω_{0y} obtained by the DDE method for the particular axis and the value of Ω_{0z} was obtained from a fit to PSD_{zz}. The parameter ξ_x was obtained from the DDE method for the x axis, Γ was calculated using the theoretical formula [5], and the temperature was the mean of T_x and T_y obtained by the DOA method. Even in the 3D case the parameters evaluated by all the methods follow the experimental trends, including the damping coefficient Γ evaluated by the DDE method, see crosses in Fig. 5 in the main text.

Considering the NP motion in the whole 3D space, the NP oscillating in one particular axis feels on average slightly weaker potential comparing to 1D case under the same potential shape along this axis. This is caused by the motion in other two axes where the NP also moves out of the beam center and therefore the optical potential is weakened. Consequently the oscillation frequency in 3D case is lower comparing to the 1D case due to the lower averaged potential. In order to quantity this effect we firstly rewrite equation (S6) in the following way:

$$F_x = -m\Omega_{0x}^2(y,z)\; x\; \exp\left(-\xi_x x^2\right), \tag{S10}$$

where

$$\Omega_{0x}(y,z) = \Omega_{0x} \exp\left(-\frac{\xi_x \Omega_{0y}^2}{2\Omega_{0x}^2} y^2\right) \exp\left(-\frac{\xi_x \Omega_{0z}^2}{2\Omega_{0x}^2} z^2\right). \tag{S11}$$

During the particle motion in the yz plane we may assume that the particle stays at a given infinitesimal interval with probability density

$$P(y,z) = \frac{1}{2\pi\sigma_y\sigma_z} \exp\left(-\frac{y^2}{2\sigma_y^2} - \frac{z^2}{2\sigma_z^2}\right), \tag{S12}$$

where σ_y, and σ_z are "effective" standard deviations of the axial harmonic motion. All methods which analyse particle trajectories in order to obtain the oscillation eigenfrequency then inherently assume that the motion between coordinate axes is decoupled and results in a value which is averaged over the extent of the axial motion. Such averaged eigenfrequency is

$$\bar{\Omega}_{0x} = \iint \Omega_{0x}(y,z) P(y,z) \mathrm{d}y\mathrm{d}z = \frac{\Omega_{0x}}{\sqrt{1+\sigma_y^2 \xi_x \frac{\Omega_{0y}^2}{\Omega_{0x}^2}} \sqrt{1+\sigma_z^2 \xi_x \frac{\Omega_{0z}^2}{\Omega_{0x}^2}}}. \tag{S13}$$

Furthermore, we can roughly estimate the value of position variance σ_i^2 using equipartition theorem for Duffing oscillator

$$m\Omega_{0i}^2\left(\sigma_i^2 + \frac{1}{2}\xi_i\langle x_i^4\rangle\right) = k_{\mathrm{B}}T. \tag{S14}$$

Since the Duffing quartic term is negligible in our case (on the order of units of percent of the quadratic term), we can approximate

$$\sigma_i^2 \simeq \frac{k_{\mathrm{B}}T}{m\Omega_{0i}^2}. \tag{S15}$$

Merging equations (S13) and (S15) we obtain for the x eigenfrequency averaged in the other two axes

$$\bar{\Omega}_{0x} = \frac{\Omega_{0x}}{1+\frac{k_{\mathrm{B}}T\xi_x}{m\Omega_{0x}^2}}. \tag{S16}$$

When we analyze the results of 3D case using above presented methods, we obtain averaged eigenfrequencies $\bar{\Omega}_{0i}$. To compare them correctly with the values Ω_{0i} that input the simulations, one should use equation (S16):

$$\Omega_{0i} = \bar{\Omega}_{0i}\left(1+\frac{k_{\mathrm{B}}T\xi_x}{m\Omega_{0x}^2}\right). \tag{S17}$$

Even though this correction is at the level of 0.5%, it provides a distinguishable shift as Fig. 5 in the main text demonstrates.

2 Extended 1D and 3D stochastic simulations

The simulations described in the previous section were repeated for a set of different input parameters to illustrate how the methods are sensitive and applicable on systems with key parameters beyond our experimental range presented in Fig. 5 in the main text.

2.1 1D simulations

We considered a 1D motion of a particle in Duffing and Gaussian profiles of the potentials with the following set of parameters:

Eigenfrequency: $\Omega_0/2\pi$ = 80 and 90 kHz

Pressure: p = 0.01, 0.1, 1, 10 and 100 mBar

Coefficient of nonlinearity: ξ = 0, 2, 5, and 10 μm^{-2}. These values are valid for eigenfrequency 90 kHz, for the other eigenfrequency (80 kHz) the coefficient of nonlinearity is scaled by the square of ratio of eigenfrequencies ($\Omega_{0y}^2/\Omega_{0x}^2$).

Temperature: T = 20, 100, and 200 °C

The simulations were performed 10 times for each combination of the input parameters (i.e. 120 combinations in total) and further analyzed by all methods described above. The trajectory simulation and its analysis was repeated 10 times for each set of input parameters and the results of those simulations were averaged and further analyzed. Fig. S1 compares the results for both types of the potential profiles where the comparison between the obtained and input values of parameters is expressed as their ratio. In order to visualize the broad parametric space the same values of parameters are encoded with the same color at the top of the Fig. S1 and plotted data points correspond to parametric set aligned vertically above them. Each curve depicting results of particular method is divided into segments:

- The shortest segment of 3 points corresponds to changing temperature T while the other parameters are fixed.
- Each of the consequent segments separated by a tiny space corresponds to changing value of coefficient of nonlinearity ξ.
- Four segments corresponding to different ξ and the same pressure are separated by a gray vertical line.

2.2 3D simulations

The motion was simulated also in 3D Gaussian potential. The forces are given by equations (S6–S9). The input parameters were $\Omega_{0x}/(2\pi)$ = 90 kHz, $\Omega_{0y}/(2\pi)$ = 80 kHz, and $\Omega_{0z}/(2\pi)$ = 25 kHz. The coefficient of nonlinearity in the x direction was ξ_x = 0, 2, 5, and 10 μm^{-2} and in the other two axes the nonlinear coefficient was scaled by the square of ratio of eigenfrequencies. The temperature and pressure values were the same as in the 1D simulations. The results of the analysis of the 3D simulated trajectories are plotted in Fig. S2 as the ratio of obtained and input parameters.

2.3 Results and discussion

Based on the performed simulations we may find the following rules for biases introduced by various methods for the following parameters:

Eigenfrequency Ω_0: All methods give correct values in case of the harmonic oscillator ($\xi_x = 0$). PSD method introduces a bias towards smaller values which is increased with the increasing nonlinearity and temperature of the system. DDE and DOA methods give correct values for a 1D oscillator while in case of the nonlinearly coupled 3D motion eigenfrequencies are biased towards lower values. However, this bias is only about $\sim 1/3$ of the bias introduced by the PSD method.

Coefficient of nonlinearity ξ: Both DOA and DDE methods give a good agreement with the input values for higher eigenfrequencies corresponding to the transversal motion of the particle. In case of 1D simulations the DDE method performs better than the DOA method while for the 3D motion the behaviour is opposite. In 3D the DOA method gives values with the deviation about 10% while the DDE method could be biased towards higher values about 50%, especially for higher values of ξ and T.

Damping coefficient Γ: Due to the asymmetry and broadening of the resonance peak the PSD method returns incorrect values for all cases including the nonlinearity both in 1D and 3D. DDE method performs well in 1D case but fails in case of the nonlinearly coupled 3D motion. However, this method still performs about 1 order of magnitude better than the PSD method. On contrary the DOA method performs extremely well both in 1D as well as in 3D cases. Only in the case of the 3D motion at low pressures the obtained value of Γ is about $2\times$ the value of the simulation input, but at these pressures the other methods differ by 2–3 orders of magnitude from the correct value.

Effective temperature T**:** The temperature analysis based on the saturated level of variance DOA results in the reasonable correspondence with the correct value (maximum error from the correct value about 20 %), while the bias is bigger for the higher temperature and the coefficient of nonlinearity. Even better results can be obtained by the frequency integration of the velocity power spectral density PSD_{vv}.

References

1. Gieseler, J., Novotny, L. & Quidant, R. Thermal nonlinearities in a nanomechanical oscillator. *Nat. Phys.* **9**, 806–810 (2013).

2. Strogatz, S. *Nonlinear Dynamics and Chaos with Applications to Physics, Biology, Chemistry and Engineering* (Westview Press, Boulder, 2015).

3. Grønbech-Jensen, N., Hayre, N. R. & Farago, O. Application of the G-JF discrete–time thermostat for fast and accurate molecular simulations. *Comput. Phys. Commun.* **185**, 524–527 (2014).

4. Yoneda, M. & Aikawa, K. Thermal broadening of the power spectra of laser-trapped particles in vacuum. *J. Phys. B: At. Mol. Opt. Phys.* **50**, 245501–9 (2017).

5. Li, T., Kheifets, S. & Raizen, M. G. Millikelvin cooling of an optically trapped microsphere in vacuum. *Nat. Phys.* **7**, 527–530 (2011).

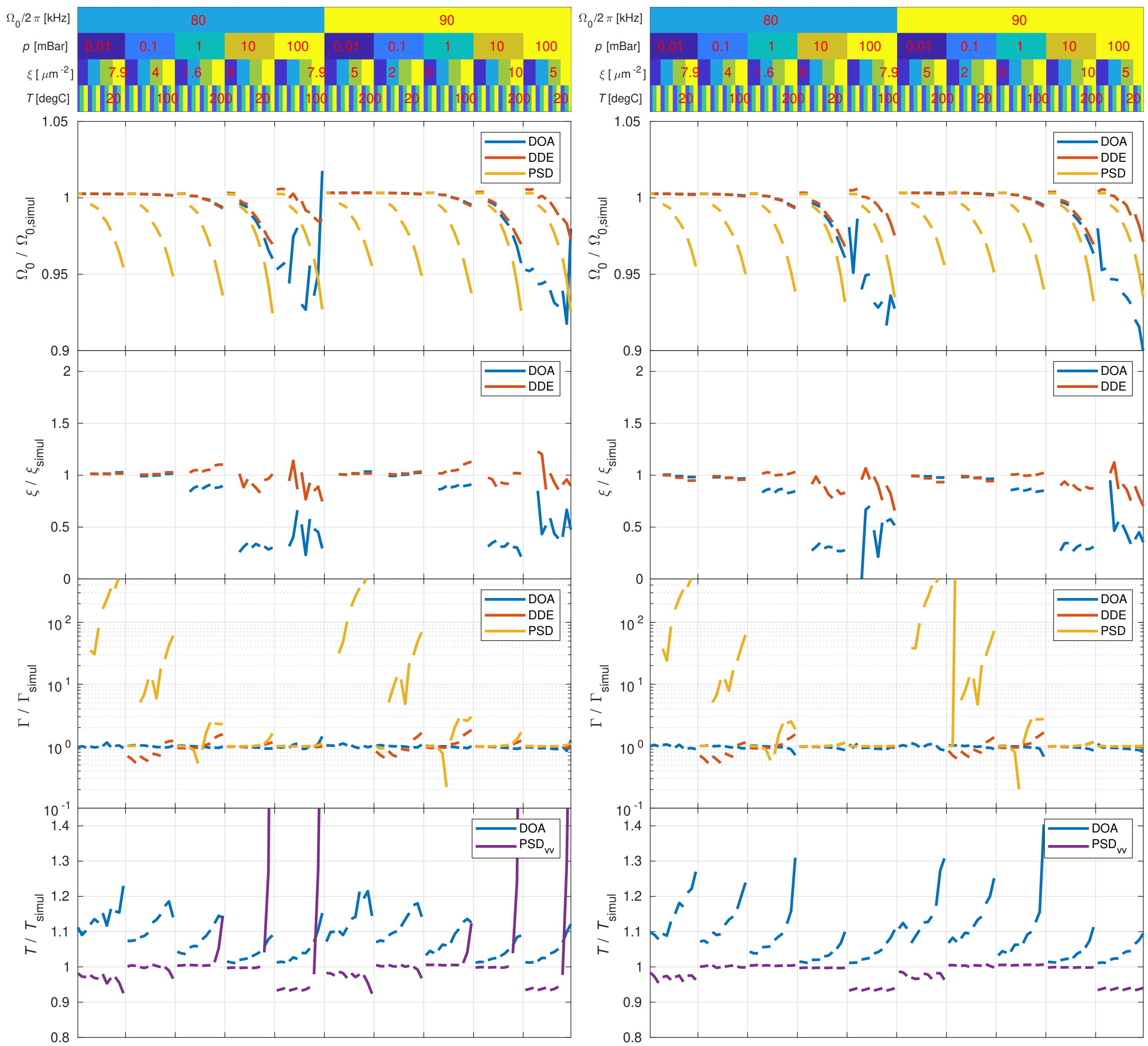

Supplementary figure S1. Relative ratio of parameters obtained by processing the 1D simulated trajectories with respect to the values of the parameters entering the simulations. For explanation of top color stripes and segments, see the text. (left) NP motion was simulated in the Duffing potential, (right) the motion was simulated in the Gaussian potential.

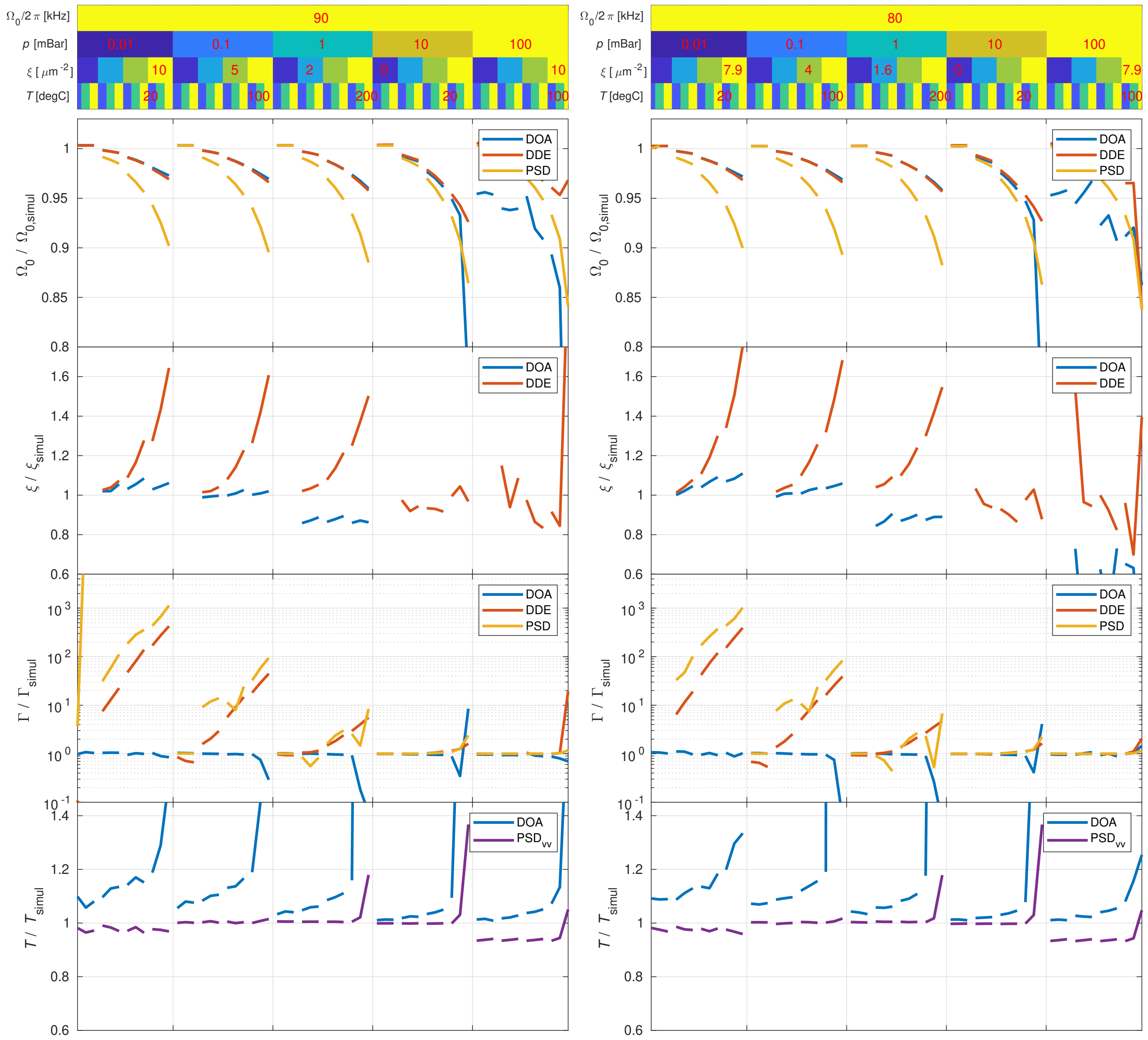

Supplementary figure S2. Relative ratio of parameters obtained by processing the 3D simulated trajectories in Gaussian potential with respect to the values of the parameters entering the simulations. For explanation of top color stripes and segments see the text. (left) result of analysis of *x* axis, result of analysis of *y* axis.
